# Investigation of Possible Factors Influencing the Neutralizing Anti-SARS-CoV-2 Antibody Titer after Six Months from the Second Vaccination Dose in a Sample of Italian Nursing Home Personnel

**DOI:** 10.3390/antib11030059

**Published:** 2022-09-19

**Authors:** Alberto Modenese, Stefania Paduano, Rossana Bellucci, Simona Marchetti, Fulvio Bruno, Pietro Grazioli, Roberto Vivoli, Fabriziomaria Gobba, Annalisa Bargellini

**Affiliations:** 1 Department of Biomedical, Metabolic and Neural Sciences, University of Modena & Reggio Emilia, 41125 Modena, Italy; 2 Laboratorio Analisi TEST SrL, 41121 Modena, Italy; 3 Fondazione Scarpari Forattini Onlus, 46020 Schivenoglia, Italy

**Keywords:** SARS-CoV-2, COVID-19, healthcare workers, vaccines, antibody titer, adverse effects, health surveillance

## Abstract

The titer of the anti-SARS-CoV-2 antibodies produced after vaccination shows a relevant decay over time, as demonstrated in several studies. However, less is known on the possible factors affecting the entity of this decay. The aim of this study is to analyze a group of individual factors which are possibly associated with anti-SARS-CoV-2 antibody titer decay six months after the second vaccine dose. We report here the results of a follow-up serological analysis and a questionnaire-based evaluation of a sample of workers from an Italian nursing home, vaccinated with two doses of BNT162b2 vaccine in early 2021. The baseline data were collected one month after the vaccine, while in the present analysis we report the data collected six months later. Our data show a relevant decay of the neutralizing antibody titer, even if for all the workers a largely positive response was detected. Moreover, our results demonstrate a possible association between younger age and the absence of previous COVID-19 infection, and a higher decay rate of the anti-SARS-CoV-2 antibodies titer.

## 1. Introduction

The decrease in the protection against SARS-CoV-2 new infections and re-infections after a few months from the second vaccine dose has highlighted the need for a third booster dose. The administration of this further anti-COVID-19 vaccine dose began in European and North American Countries in the second half of the year 2021 [1,2,3,4]. Currently, a fourth vaccine dose for subjects who are particularly at risk of infection and its severe consequences has been proposed [5,6]. Since the beginning of the COVID-19 pandemic, one of the ways to evaluate the presence of an immune response to SARS-CoV-2, acquired after a previous infection or after the vaccination, was the determination of serological anti-SARS-CoV-2 antibody titer [1,2,3,4,5,6]. It is now known that these antibodies only represent a component of the immune response, and that the direct role of memory T cells in killing virus-infected cells is equally fundamental [7]. Nevertheless, the presence of neutralizing antibodies against SARS-CoV-2 is still an important indicator used to determine whether a subject might have a positive response to the vaccine, and their presence is also evaluated in some studies aimed at exploring possible associations with COVID-19 symptoms and prognosis [1,2,3,4,5,6,8,9]. The main target of the anti-SARS-CoV-2 neutralizing antibodies investigated after the performance of the vaccination is the spike (S) protein, and in particular its Receptor-binding Domain (RBD) [10]. Several scientific reports show a rapid decrease of the IgG anti-RBD titer a few months after the vaccination, but there is still a lack of knowledge about the possible factors that may play a role in influencing this decay [1,2,3,4,5,6,11]. As various conditions are currently considered associated with an increased likelihood of contracting a severe illness from COVID-19 [12], the hypothesis of this research is that there may also be possible factors influencing the anti-SARS-CoV-2 antibody titer. These factors are: (i) older age; (ii) the number of underlying medical conditions, including (but not limited to) cancer, chronic kidney disease, liver disease, chronic respiratory diseases, various neurological conditions, diabetes, cardiovascular diseases including hypertension and various others; (iii) long-standing systemic health and social inequities, certain ethnic minority groups and disabilities; (iv) a smoking habit; (v) pregnancy; (vi) refusal of, or contraindication to undergo, anti-COVID-19 vaccination; (vii) inadequate compliance with the preventive measures for COVID-19 [12]. Considering the diseases, certainly those of them directly affecting the immune response and causing immunodeficiency (e.g., HIV infection, lymphoproliferative and immunological diseases and others) or those conditions requiring chronic immunosuppressive therapy (e.g., use of antiblastic drugs, radiotherapy, prolonged use of corticosteroids and others) can both have an influence on the antibody titer [12,13,14]. Another factor of interest from an occupational health point of view for healthcare workers and other professions is the performance of nightshifts at work. This is a recognized work-related risk factor, possibly involved in the dysregulation of the circadian rhythm with repercussions also for the immune function [13,15]. Considering the relation with SARS-CoV-2 infection, it has been reported that nightshift healthcare workers during the pandemic had a higher job demand, longer working hours and poorer sleep quality, therefore increasing the occasions for contagion [13,16,17,18]. Nevertheless, the possible dysregulation of the circadian rhythm influencing the preparedness of the immune system for responding to the infections cannot be ruled out [15]. Moreover, it should also be underlined that published data suggest a significant detection of lower vitamin D levels [19] and higher Body Mass Index (BMI) [16] in nightshift workers: both these conditions have been reported as associated with an increased risk of COVID-19 [16,17,18,20,21]. Finally, another condition that was specifically considered among our hypothesis of factors possibly influencing the anti-SARS-CoV-2 antibodies level after the anti-COVID-19 vaccination, and its decay after six months, was the recent performance of other vaccination(s), and in particular of anti-influenza vaccination. The rationale is that another vaccine could possibly provide a certain degree of cross-protection against other viral infections [22,23].

According to these premises, the objective of this work is to evaluate a series of individual factors which are possibly associated with a significant decrease of the anti-RBD IgG antibody titer in a sample of healthcare workers (HCW) six months after the second dose of the COVID-19 BNT162b2 vaccine.

## 2. Materials and Methods

### 2.1. Description of the Study

We performed a new evaluation of a previously described group of workers employed in a nursing home in northern Italy, who received two doses of the anti-SARS-CoV-2 BNT162b2 vaccine in early 2021 [24]. The nature of the study is observational: after six months from the baseline investigation, we returned to the same north-Italian small nursing home, proposing to the workers a further cross-sectional follow-up investigation, including the collection of a blood sample for the determination of anti-SARS-CoV-2 antibodies and the participation to a self-reported survey, administered on paper.

The inclusion criteria of this follow-up study were:

- being an employee of the nursing home at work during the period January–August 2021;
- being vaccinated with two doses of the Pfizer/BioNTech BNT162b2 anti-SARS-CoV-2 mRna vaccine between 12 January and 17 February of 2021 (i.e., the dates that we identified during the baseline investigation);
- having participated to our first baseline investigation, with an available anti-SARS-CoV-2 IgG antibody titer determined via a serological test performed in our lab.

No other exclusion criteria were applied.

We did not only consider healthcare operators for our analysis, but also included other workers employed in the nursing home such as administrative personnel, cleaning service and kitchen staff and maintenance workers. Healthcare workers (HCW) were categorized as follows: nurses, nurses’ assistants, physicians and other HCW (e.g., physiotherapists). All the personnel voluntarily agreed to participating in our study, and signed a written informed consent. The study was approved by our local institutional review board.

As reported in our prior publication [24], four weeks after the second dose of the vaccine, a blood sample was collected from all the subjects to determine the titer of IgG-neutralizing anti-RBD antibodies of the subunit S1 of the spike protein of SARS-CoV-2. With the present analysis, we consider a further determination of these IgG performed six months after the second dose. The test applied was the same for the first and the second antibody titer determination, i.e., the EUROIMMUN Anti-SARS-CoV-2 QuantiVac ELISA (IgG) test, with the interpretation of the results in Binding Antibody Units per milliliter (BAU/mL), considering as negative a titer < 26 BAU/mL, positive a titer ≥ 36 BAU/mL, while a titer between 26 and 35 BAU/mL [25,26] was of doubtful interpretation.

During the baseline investigation we collected a self-reported questionnaire [24] (Supplementary Materials File S1) and a second questionnaire, which was similar but shorter, was collected for the follow-up analysis to register any relevant updated information (Supplementary Materials File S2). The survey was distributed on the occasion of the collection of the blood sample for the determination of the antibodies level. All the information that was collected with the two questionnaires is fully reported in the Supplementary Materials (Files S1 and S2). In the present analysis, we specifically considered the following variables: sex, age, body mass index (BMI), smoking habit, job category, performance of nightshifts at work, performance of anti-influenza vaccine at the end of 2020/beginning of 2021, date of COVID-19 diagnosis—reported number, if any, of concomitant diseases. This last variable was obtained based on the answers to open questions (see Files S1 and S2), coded ex-post by the researchers (A.M. and S.P.) in order to build the variable “pathologies number”, categorized as 0, 1 or ≥2.

### 2.2. Statistical Analysis

Logarithmic transformation was used to normalize anti-SARS-CoV-2 neutralizing IgG and the results are expressed as median and ranges. The chi-square test, paired *t* test, and one-way analysis of variance (ANOVA) with the Bonferroni test were applied whenever necessary. Mann–Whitney test was used to compare the percentage of anti-SARS-CoV-2 neutralizing IgG titer decline between two subgroups and Kruskal–Wallis test for the comparison of three or more subgroups. Multivariate linear regression analysis was performed to determine whether the participants’ characteristics (age, sex, previous SARS-CoV-2 infection, BMI, smoking status, job category, nightshifts at work, number of concomitant diseases and previous anti-influenza vaccine) influenced anti-SARS-CoV-2 neutralizing IgG titer decline six months after the vaccination. Data collected were analyzed using STATA Software (release 15, StataCorp, College Station, TX, USA). Regarding statistical significance, a *p* value < 0.05 was considered.

## 3. Results

### 3.1. Characteristics of the Study Population and Anti-SARS-CoV-2 Antibody Titer in the Vaccinated Subjects after One Month and Six Months from the Second Vaccine Dose

A total of 63 out of 74 employees of the nursing home (85.1%) investigated in our first study agreed to participate in the follow-up and gave a further blood sample for the determination of the anti-SARS-CoV-2 IgG titer. Workers were for the 79.4% females, with a median age of 54 years old (range: 25–73). Regarding job type, 44.5% of the sample was composed of nurses, nurses’ assistants and physicians (*n* = 28), 20.6% by other HCWs (*n* = 13), 14.3% by kitchen and cleaning personnel (*n* = 9) and 20.6% by technical and administrative personnel (*n* = 13).

**Figure 1:**
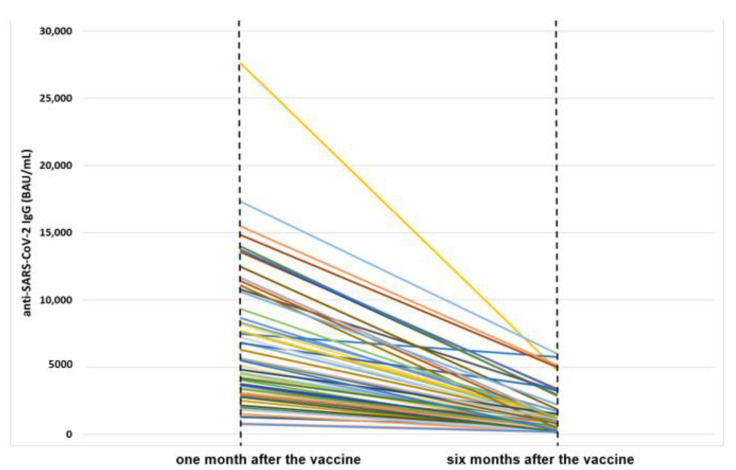
Decrease in the anti-SARS-CoV-2 neutralizing IgG titer: trend of the levels detected one month vs. six months after the anti-COVID-19 BNT162b2 vaccine, graphically represented with a line for each of the 63 workers of the nursing home included in the study sample.

The median IgG titer one month after the vaccination was 4821 BAU/mL (range: 764–27,600), while after six months the titer was 876 (156–6034). For all the subjects, a decrease of the titer was observed and the difference between the first and the second determination was highly significant (*p* < 0.001), also considering the subjects grouped according to the different variables investigated (i.e., sex, age classes, smoking habit, BMI, previous COVID-19 diagnosis, anti-influenza vaccination and number of concomitant diseases) (Table 1). In Figure 1 we show a visual description of the decrease in the antibody titer for the 63 workers (Figure 1).

### 3.2. Evaluation of the Decrease of Anti-SARS-CoV-2 Antibody Titer in the Vaccinated Subjects Six Months after the Second Dose and of the Possible Associated Factors

In Table 1 we report the median values and ranges of neutralizing antibodies against SARS-CoV-2, measured 6 months after two doses of BNT162b2 vaccine in the 63 nursing home workers included in the follow-up analysis, evaluated according to various characteristics of the population.

Overall, the decline of the antibody titer reached 83%, slightly higher in males compared to females (84.5 vs. 82%), even if the difference is not statistically significant (Table 1).

We observed a significant difference evaluating the decay of the titer with respect to age-classes: in the younger group, the highest decrease of the titer was observed (i.e., <91%), while in the older group the decrease of 78% is lower compared to the other age-classes (*p* = 0.04, Table 1). In the linear regression analysis, age resulted in a coefficient of −4.58 (CI 95% −7.25–−1.91, *p* = 0.001).

No significant differences in the decrease of neutralizing antibodies’ titer were observed according to BMI and smoking habit, and, as expected, according to job type (Table 1). A lower percentage reduction of the antibody titer, even if not statistically significant, was found when comparing workers performing nightshifts at work vs. those not performing nightshifts (80.5 vs. 84.5%, *p* = 0.085) (Table 1). A significantly higher percentage decrease in the antibody titer is reported for the workers who previously underwent an anti-influenza vaccination (*p* = 0.017), as well as for those with no previous COVID-19 infection (*p* = 0.016) (Table 1). Using multivariate linear regression analysis, previous anti-influenza vaccination resulted in a coefficient of 7.33 (CI 95% 1.02–13.64, *p* = 0.024), while for previous COVID-19 the coefficient was −2.57 (CI 95% −13.30–−1.63, *p* = 0.013).

Considering the number of concomitant diseases reported, no differences in the reduction of the titer were identified when comparing workers with no diseases vs. personnel with 1 or ≥2 pathologies (Table 1).

## 4. Discussion

Our results are a further confirmation of the relevant decline of the anti-SARS-CoV-2 IgG titer six months after the second dose of anti-COVID-19 vaccination [1,2,3,4,5,6]. Nevertheless, at the same time, the results we obtained represent also a further demonstration that, six months after completing the vaccination, there is still a positive antibody response, possibly indicating a protective effect [27,28,29], which in our case was identified in 100% of the sample. In fact, the IgG titer remained indicative of a positive neutralizing antibody response (i.e., according to the manufacturer ≥ 36 BAU/mL, for all the 63 subjects, even after six months from the second dose). The antibodies’ titers we detected in the sample observed of workers of a nursing home had a relevant decline of −83% after six months, confirming that a booster vaccine dose was needed: HCW in Italy, together with the fragile population, were the first groups to receive the third vaccination dose, since the end of September 2021 [30]. This choice was determined by the fact that HCW have an increased COVID-19 risk [31,32], and also that they were the first group who received the vaccination at the beginning of the campaign in early 2021 (and therefore a longer period had elapsed since the completion of the vaccination cycle, possibly resulting in a relevant decline of the neutralizing antibody titer).

The second objective of this work was to identify possible factors associated with different decay rates of anti-SARS-CoV-2 IgG titer six months after the vaccines. It should be underlined that, considering our data, the most relevant decline was observed in those subjects who had a higher antibody titer at the baseline, confirming recent data [23]. This is particularly clear when we look at our study sample grouped according to age classes: our results show that the highest decline was identified in the youngest group, while the lowest was in workers who are over 60. As mentioned above, this is most likely related to the fact that subjects ≤ 30 years of age were those with the highest antibody titer compared to all the other age classes one month after the vaccination, showing a median titer of 8288 BAU/mL. A possible exception to this interpretation is the difference observed in antibody titer considering gender: females start with a higher antibody titer compared to males, confirming previous data [13]. Nevertheless, the percentage decline we measured is lower in females than in males (−82.1 vs. −84.5%), even if the difference is not significant.

With regards to the role of previous SARS-CoV-2 infection, it is well documented that HCW who had a SARS-CoV-2 infection plus a vaccination reach higher anti-SARS-CoV-2 antibody titer one month after the vaccination when compared to subjects with no infection history, and our results are in agreement with these findings [11,24,28]. Moreover, we found that these subjects with previous infection reported a significantly lower decay of the titer six months later.

Considering recognized risk factors for COVIID-19 as high BMI, smoking status and the number of concomitant diseases [12], we did not observe significant differences in the decline of the neutralizing antibody response when comparing the titers one vs. six months after the vaccination. Finally, of doubtful interpretation is the result related to anti-influenza vaccination, as we observed a significantly lower decline in the antibody titer in subjects who did not report a influenza vaccine at the end of 2020/beginning of 2021 compared to those who reported this vaccination. These data represent only a preliminary finding suggesting an association, but it has to be interpreted with caution, unless it is confirmed in adequately designed studies, with a sufficiently large sample size. To the best of our knowledge, this is the first report of a higher decay of the anti-SARS-CoV-2 IgG titer among subjects with recent anti-flu vaccination. We found a similar observation also in the Greco et al. article: in this study the group that reported anti-influenza vaccine had a higher decay, but they started from a higher antibodies level in both the first and the second determination compared to the non-vaccinated subjects [23]. In our case, we tested various hypotheses in an attempt to explain the slightly significant association we found, and we particularly focused on the possible effects of the age classes and of all the other factors we investigated. We did not find any significant difference in anti-influenza self-reported vaccination rates across age groups, and the statistical difference we calculated also remained valid after adjustments in the multivariate linear regression analysis. As further stressed below in the discussion of our study limitations, we believe that the main reasons of this finding do not depend on a real causal association, but most likely on the effect determined by the exceptional contribution of single individuals able to influence the overall results, due to the small sample size.

Among the main limitations of our study there is the small sample size and the observational nature of the research, analyzing self-reported data collected with a survey. Among all the variables analyzed, the self-reporting may have been particularly critical for the variables related to the medical anamnesis of the subjects, including the data on anti-flu vaccination and those on concomitant diseases. This data would be better captured with more objective methods, based on the direct collection of clinical data by health professionals. Regarding the diseases, we tried to overcome this limitation by performing a blind and rigorous ex-post coding of the answers to the open questions given by the respondents. Moreover, we tested various approaches, categorizing the diseases in different ways, considering the specific types of pathologies, finally keeping the most reliable variable we used in the multivariate analysis (i.e., the number of concomitant diseases of any type). Regarding the anti-flu vaccine, in this case the possibility of further cleaning and testing the self-reported data in different ways was not reliable, as we had only one yes/no question based on the survey administered. For this reason, the finding related to anti-influenza vaccine must be interpreted with extra caution, and has to be confirmed with an additional analysis of larger samples, possibly including more objective data on the performance of the vaccine. Our study may have also technical limitations, referred in particular to the determination of the anti-SARS-CoV-2 antibody titers and to the assay performed, including the possible presence of different immunoglobulins’ isotopes and their relations with the total level of circulating antibodies. Moreover, other limitations of our study are intrinsically related to its nature and to the source population, which is selected from a small nursing home located in northern Italy, where the spread of SARS-CoV-2 was particularly relevant compared to other areas of Italia and the world during the first few months of the pandemic. Furthermore, it should be taken into consideration that this is a professionally exposed population, with a significantly higher exposure to SARS-CoV-2 compared to the general public: this peculiarity, together with the already mentioned issue of the small sample size, makes the extrapolation of our findings to a wider public not possible. Finally, the limitations intrinsically related to the variability over time of the virus, and in particular of SARS-CoV-2, should also be mentioned here: the changes of the virus, considering the quite long duration of our study, may have influenced the antibodies’ titer of our population, with different individual responses based on the virus variant and viral load exposure.

Despite these limitations, our study has also important strengths, as almost all the subjects investigated one month after the vaccination agreed to participate again six months later, filling in the same questionnaire administered the first time and being evaluated for the anti-SARS-CoV-2 with the same test applied for the first serological determination. Moreover, all the subjects had been vaccinated with two doses of the same anti-COVID-19 vaccine, indicating that the possible differences in the decline of the antibodies’ titers, identified across sub-groups, cannot be related to different vaccines used or different number of doses administered.

## 5. Conclusions

Our study in a sample of Italian nursing home workers shows a relevant decay of anti-SARS-CoV-2 neutralizing antibodies six months after the second BNT162b2 vaccine dose. A largely positive antibody response was detected in the totality of the sample. We found that the absence of previous COVID-19 infection and a younger age were significantly associated with a higher decay of the antibody titer.

## Figures and Tables

**Table 1:** Titer (BAU/mL, median and range values) and titer decay (∆, %) of neutralizing anti-SARS-CoV-2 antibodies, measured 1 vs. 6 months after the second dose of the BNT162b2 vaccine in 63 workers of an Italian nursing home, grouped according to the main individual and occupational characteristics.

		N (%) *	Anti-SARS-CoV-2 IgG (BAU/mL) One Month after Vaccination–MD (Range: Min–Max)	Anti-SARS-CoV-2 IgG (BAU/mL) Six Months after Vaccination—MD (Range: Min–Max)	*p*-Value	∆ Anti-SARS-CoV-2 IgG (One vs. Six Months after Vaccination) BAU/mL MD (Q1; Q3)	% (Anti-SARS-CoV-2 IgG) Decline Six Months after Vaccination MD (Q1; Q3)	*p*-Value
Overall		63 (100)	4821 (764–27,600)	876 (156–6034)	<0.001	3802 (2465; 7367)	82.9 (78.5; 88.6)	
Sex	Males	13 (20.6)	3398 (764–17,300)	355 (161–6034)	<0.001	2870 (1790; 7367)	84.5 (78.9; 91.0)	0.445
	Females	50 (79.4)	5285 (830–27,600)	895 (156–5748)	<0.001	4074 (2472; 7010)	82.1 (78.5; 87.1)	
Age class ^a^	≤30	6 (9.5)	8288 (3695–13,749)	756 (239–3347)	<0.001	7783 (4472; 10,402)	91.3 (81.2; 94.9)	0.041
	31–40	11 (17.5)	3074 (1824–11,390)	355 (169–1398)	<0.001	2452 (1789; 3426)	83.4 (81.8; 91.0)	
	41–50	12 (19.1)	4820 (2508–12,465)	882 (245–2242)	<0.001	4074 (2623; 5473)	83.6 (80.5; 86.7)	
	51–60	21 (33.3)	4821 (830–15,474)	1035 (156–5748)	<0.001	3280 (2627; 6556)	84.2 (74.9; 85.9)	
	>60	13 (20.6)	6783 (764–27,600)	1293 (161–6034)	<0.001	5034 (3085; 9811)	78.5 (69.9; 82.5)	
BMI	<25	32 (51.6)	4700 (830–13,749)	668 (169–3347)	<0.001	3844 (2438; 5817)	83.6 (79.5; 89.9)	0.208
	25–29.9	25 (40.3)	6783 (764–17,300)	1169 (161–6034)	<0.001	3372 (2472; 7540)	80.2 (73.5; 85.1)	
	≥30	5 (8.1)	4557 (2783–14,834)	588 (156–5024)	<0.003	3969 (2870; 6285)	84.5 (81.8; 87.1)	
Smoking habit	Non-smokers	48 (76.2)	4568 (764–27,600)	669 (156–6034)	<0.001	3752 (2462; 8197)	82.7 (78.7; 88.4)	0.796
	Smokers	15 (23.8)	6783 (1824–13,551)	1205 (175–5748)	<0.001	3986 (2472; 6556)	83.0 (74.9; 88.6)	
Job type	HCW	28 (44.4)	6226 (1504–27,600)	1163 (169–6034)	<0.001	4355 (3018; 8926)	82.3 (74.2; 86.0)	0.407
	Other employees	35 (55.6)	4150 (764–15,474)	588 (156–5174)	<0.001	3456 (2452; 6285)	83.0 (79.1; 89.1)	
Nightshifts at work	No	49 (80.3)	4254 (764–17,300)	588 (156–6033)	<0.001	3456 (2465; 7367)	84.5 (79.1; 89.1)	0.085
	Yes	12 (19.7)	5959 (2508–27,600)	1221 (245–5748)	<0.001	4074 (2448; 6493)	80.5 (75.7; 83.3)	
Anti-influenza vaccine	No	39 (61.9)	5062 (830–27,600)	1035 (175–5748)	<0.001	3702 (2404; 8354)	81.2 (75.1; 85.0)	0.017
	Yes	24 (38.1)	4527 (764–17,300)	558 (156–6034)	<0.001	3886 (2811; 5879)	86.6 (79.4; 92.0)	
COVID-19 diagnosis	No	34 (54.0)	3721 (764–27,600)	512 (156–5174)	<0.001	3310 (2452; 6556)	85.2 (79.3; 91.5)	0.016
	Yes	29 (46.0)	6783 (1310–17,300)	1205 (178–6034)	<0.001	5034 (2870; 9811)	81.2 (73.5; 85.0)	
Pathologies number	0	32 (50.8)	4364 (830–15,474)	759 (175–5174)	<0.001	3752 (2438; 6720)	82.6 (79.0; 87.3)	0.420
	1	22 (34.9)	5695 (764–27,600)	540 (161–6034)	<0.001	3679 (2535; 8041)	85.2 (78.9; 88.7)	
	≥2	9 (14.3)	4821 (2783–14,834)	1169 (156–5024)	<0.001	3969 (3085; 6285)	79.0 (72.5; 85.0)	

MD = median, BAU/mL (binding antibody units per milliliter), HCWs (healthcare workers), BMI (body mass index). * The percentages were calculated excluding missing values. ^a^ Bonferroni: *p* = 0.031 between age class 31–40 vs. >60, no significant difference between other categories.

## Data Availability

The data presented in this study are available on request from the corresponding author. The data are not publicly available due to restrictions for reasons of privacy and ethics.

## Supplementary Materials

The following supporting information can be downloaded at: https://www.mdpi.com/article/10.3390/antib11030059/s1, File S1: Baseline questionnaire for the study “Antibody response after anti-SARS-CoV-2 vaccination in healthcare operators and possible associated factors”; File S2: Follow-up questionnaire for the study “Antibody response after anti-SARS-CoV-2 vaccination in healthcare operators and possible associated factors”.
