# Supplementary material for: Investigation of Possible Factors Influencing the Neutralizing Anti-SARS-CoV-2 Antibody Titer after Six Months from the Second Vaccination Dose in a Sample of Italian Nursing Home Personnel
Source: Antibodies (Basel). 2022 Sep 19;11(3):59. doi: 10.3390/antib11030059 (PMC9495803; doi:10.3390/antib11030059)
Supplement: Supplementary file 1 [file antibodies-11-00059-s001.zip › antibodies-1838878-supplementary.pdf]

File S1: baseline questionnaire for the study "Antibody response after anti-SARS-CoV-2 vaccination in healthcare operators and possible associated factors"

DATE [___][___] / [___][___]/ [___][___][___][___]

Gender M F Other

Date of birth ____

Country of birth ____

Height ___________________ Weight__________________

ANTI-COVID-19 VACCINATION: NO YES

If yes, DATE 1° DOSE [___][___] / [___][___]/ [___][___][___][___]

If yes, DATE 2° DOSE [___][___] / [___][___]/ [___][___][___][___]

TYPE OF ANTI-COVID-19 VACCINE:

 COMIRNATY (Pfizer-BioNtech) Moderna Astra-Zeneca

 Other: ……………………………………………………………………..

HEALTH

Are you a current smoker? NO YES If yes, since how many years? _____ n° cigarettes/day_________

Do you usually drink alcohol? NO YES
If YES, how frequently on a weekly basis do you take 1 o 2 alcoholic units per day? ____________ only occasionally And how frequently on a weekly basis do you take 3 or more alcoholic units per day? __________________ only occasionally
(please note: 1 alcoholic unit is a standard glass of wine – 0.25 l - or a small glass of spirit – 0.10 l - or a medium glass of beer – 0.4 l)

Are you affected by diabetes? **NO** **YES**

Are you affected by respiratory diseases? **NO** **YES**
If YES, which disease(s)? ____

Are you affected by cardiovascular diseases? **NO** **YES**
If YES, which disease(s)? Hypertension other cardiovascular disease(s)_________________ ____

Are you affected now or were you affected by neoplastic diseases? □ **NO** □ **YES**
If YES, which disease(s)? ______________________________If yes, when did you undergo the last therapy for this/these disease(s) ______________________________
Are you affected by other diseases? □ **NO** □ **YES**
If YES, which disease(s)? ______________________________

Do you usually assume drugs for any of all the disease(s) mentioned above (if any)? □ **NO** □ **YES**
If YES, which drug(s)? ______________________________

In general, during the last year did you take any drug(s) for a long time (at least more than 2 weeks)? □ **NO** □ **YES**
If YES, which drug(s)? for how long? ______________________________

Do you remember your latest vaccination before the anti-COVID-19 one? □ **NO** □ **YES**
If YES, which vaccination? ____________________ When were you vaccinated? __________

Did you experience any of the following symptoms during the last year?
□ cough □ cold □ sore throat □ tiredness □ diffuse joint/muscle pains □ slightly increased body temperature (about 37.5-38°C) □ highly increased body temperature (>38°C)
□ impossibility/difficulty to recognize: □ tastes □ smells □ other symptoms which I believe can be relevant with respect to COVID-19: ______________________________
____

Were you diagnosed with COVID-19 (please note: we mean that you had a confirmed positive diagnostic swab) during the last year? □ **NO** □ **YES** If YES, do you remember when? (date) ____________________
And when did you get the negative swab and/or you were confirmed as negative for COVID-19? ______________________________

Before today, and before the anti-COVID-19 vaccination, did you have at least one serological test to evaluate the presence of anti-SARS-CoV-2 antibodies IgG and/or IgM during the last year?
□ **NO** □ **YES**
If YES, do you remember the latest test you had with a negative result (i.e. with no or insufficient detection of antibodies)? (date) __________________ □ never had a negative serological test
If YES, do you remember the latest test you had with a positive result (i.e. with detection of antibodies)? (date) ____________________ □ never had a positive serological test

In case you were diagnosed with COVID-19, was this diagnosis reported to the national workers' compensation authority as a work-related infection? □ **NO** □ **YES** If YES, do you remember when? (date) ____________________

In case you were diagnosed with COVID-19 and had symptoms, did you had to undergo oxygen support therapy for respiratory fatigue? □ **NO** □ **YES**

In case you were diagnosed with COVID-19 and had symptoms, were you hospitalized?
□ **NO** □ **YES** If YES, how long have you been to the hospital? ____________________

WORK

JOB TASK ______________________________

Does your job include shifts? □ NO □ YES If YES, do you perform also night shifts? □ NO □ YES

If you perform nightshifts, how many of them did you perform during the last year?

 less than 20 21-40 41-60 61-80 81-100 or more

Due to your job, did you assist COVID-19 patients during the last year? **NO** **YES**

During the last year, did you follow specific training for the prevention of the COVID-19 risk at work? **NO** **YES**

Due to your job, did you experience any occupational injury at risk for the transmission of an infectious agent (e.g. needle stick injury) during the last year and consequently you had to undergo a profilactic therapy for the prevention of: HIV infection **NO** **YES** HBV infection **NO** **YES** TETANUS infection **NO** **YES**

During the last year, how frequently did you use the following Personal Protective Equipment at work:

DPI	NEVER	SELDOM	SOMETIMES	OFTEN	ALWAYS
Surgical mask					
Protective respiratory mask (type FFP2/N95 and/or FFP3/N98)					
Disposable gloves (One pair at a time)					
Disposable gloves (Two pairs at a time)					
Waterproof overcoat					
Overshoes					
Protective glasses					
Visor					
Protective headgear					
Other 1 (please indicate here ____ ______________________________) ____________________________					
Other 2 (please indicate here ____ ______________________________) ____________________________					

File S2: follow-up questionnaire for the study “Antibody response after anti-SARS-CoV-2 vaccination in healthcare operators and possible associated factors "

DATE [___][___] / [___][___]/ [___][___][___][___]

Gender M F Other

Date of birth ____________________ Country of birth ______________________________

ANTI-COVID-19 VACCINATION: **NO** **YES**

If yes, DATE 1° DOSE [___][___] / [___][___]/ [___][___][___][___]

If yes, DATE 2° DOSE [___][___] / [___][___]/ [___][___][___][___]

TYPE OF ANTI-COVID-19 VACCINE: COMIRNATY (Pfizer-BioNtech) Moderna Astra-Zeneca
□ Johnson & Johnson □ Other: ……………………………………………………………………..

HEALTH
During the last 6 months (i.e. after our previous investigation and the anti-COVID-19 vaccination) had you any new diagnosis for disease(s) other than COVID-19? □ **NO** □ **YES**
If YES, which disease(s)? ____

Did you experience any of the following symptoms during the last 6 months (i.e. after our previous investigation and the anti-COVID-19 vaccination)? □ cough □ cold □ sore throat □ tiredness □ diffuse joint/muscle pains □ slightly increased body temperature (about 37.5-38°C) □ highly increased body temperature (>38°C) impossibility/difficulty to recognize: □ tastes □ smells □ other symptoms which I believe can be relevant with respect to COVID-19:
____

Were you diagnosed with COVID-19 (please note: we mean that you had a confirmed positive diagnostic swab) during the last 6 months (i.e. after our previous investigation and the anti-COVID-19 vaccination)? □ **NO** □ **YES** If YES, do you remember when? (date) ____________________

If YES, can you estimate when, taking as a reference the latest vaccine dose before the infection, were you diagnosed with COVID-19? (please indicate here how many days after the first/second vaccine dose)____

If YES, was the condition you experience with COVID-19 this time a symptomatic one? □ **NO** □ **YES** If YES, which symptom(s)? ____
In case you were diagnosed with COVID-19 during the last 6 months (i.e. after our previous investigation and the anti-COVID-19 vaccination), did you had to undergo oxygen support therapy for respiratory fatigue? □ **NO** □ **YES**

In case you were diagnosed with COVID-19 during the last 6 months (i.e. after our previous investigation and the anti-COVID-19 vaccination), were you hospitalized?
□ **NO** □ **YES** If YES, how long have you been to the hospital? _____________________
